# Supplementary material for: Hepatocellular Carcinoma Recurrence and Mortality Rate Post Liver Transplantation: Meta-Analysis and Systematic Review of Real-World Evidence
Source: Cancers (Basel). 2022 Oct 19;14(20):5114. doi: 10.3390/cancers14205114 (PMC9599880; doi:10.3390/cancers14205114)
Supplement: Supplementary file 1 [file cancers-14-05114-s001.zip › cancers-1845274-supplementary.pdf]

Table S1a: PubMed search strategy

S. No.	Query	Items Found	Description
#1	"liver neoplasms"[MeSH] OR ("biliary tract" OR "hepatobiliary" OR "liver") AND (cancer* OR carcinom* OR neoplasm* OR tumor* OR sarcoma* OR tumour*) OR "ascites hepatomas" OR "hepatoblastomas" OR "liver carcinogenesis" OR "morris hepatomas" OR "novikoff hepatomas"	3,81,354	**Search terms:** Hepatocellular Carcinoma
#2	Liver transplantation	82,751	**Search terms:** Liver transplantation
#3	Recurrence rate OR Survival Rate	2,98,765	**Search terms:** Recurrence rate and Survival rate
	#1 AND #2 AND #3	2,513	**Combine search terms**

Table S1b: Epistemonikos search strategy

S. No.	Query	Items Found	Description
#1	(title:((title:(liver neoplasms) OR abstract:(liver neoplasms)) OR (title:(biliary tract cancer) OR abstract:(biliary tract cancer)) OR (title:(biliary tract carcinoma) OR abstract:(biliary tract carcinoma)) OR (title:(biliary tract tumor) OR abstract:(biliary tract tumor)) OR (title:(biliary tract sarcoma) OR abstract:(biliary tract sarcoma)) OR (title:(biliary tract tumour) OR abstract:(biliary tract tumour)) OR (title:(hepatobiliary cancer) OR abstract:(hepatobiliary cancer)) OR (title:(hepatobiliary carcinoma) OR abstract:(hepatobiliary carcinoma)) OR (title:(hepatobiliary tumor) OR abstract:(hepatobiliary tumor)) OR (title:(hepatobiliary sarcoma) OR abstract:(hepatobiliary sarcoma)) OR (title:(hepatobiliary tumour) OR abstract:(hepatobiliary tumour)) OR (title:(Liver cancer) OR abstract:(Liver cancer)) OR (title:(Liver carcinoma) OR abstract:(Liver carcinoma)) OR (title:(Liver tumor) OR abstract:(Liver tumor)) OR (title:(Liver		**Search terms:** Hepatocellular Carcinoma

	sarcoma) OR abstract:(Liver sarcoma)) OR (title:(Liver tumour) OR abstract:(Liver tumour)) OR (title:(ascites hepatomas) OR abstract:(ascites hepatomas)) OR (title:(hepatoblastomas) OR abstract:(hepatoblastomas)) OR (title:(liver carcinogenesis) OR abstract:(liver carcinogenesis)) OR (title:(morris hepatomas) OR abstract:(morris hepatomas)) OR (title:(novikoff hepatomas) OR abstract:(novikoff hepatomas))) OR abstract:((title:(liver neoplasms) OR abstract:(liver neoplasms)) OR (title:(biliary tract cancer) OR abstract:(biliary tract cancer)) OR (title:(biliary tract carcinoma) OR abstract:(biliary tract carcinoma)) OR (title:(biliary tract tumor) OR abstract:(biliary tract tumor)) OR (title:(biliary tract sarcoma) OR abstract:(biliary tract sarcoma)) OR (title:(biliary tract tumour) OR abstract:(biliary tract tumour)) OR (title:(hepatobiliary cancer) OR abstract:(hepatobiliary cancer)) OR (title:(hepatobiliary carcinoma) OR abstract:(hepatobiliary carcinoma)) OR (title:(hepatobiliary tumor) OR abstract:(hepatobiliary tumor)) OR (title:(hepatobiliary sarcoma) OR abstract:(hepatobiliary sarcoma)) OR (title:(hepatobiliary tumour) OR abstract:(hepatobiliary tumour)) OR (title:(Liver cancer) OR abstract:(Liver cancer)) OR (title:(Liver carcinoma) OR abstract:(Liver carcinoma)) OR (title:(Liver tumor) OR abstract:(Liver tumor)) OR (title:(Liver sarcoma) OR abstract:(Liver sarcoma)) OR (title:(Liver tumour) OR abstract:(Liver tumour)) OR (title:(ascites hepatomas) OR abstract:(ascites hepatomas)) OR (title:(hepatoblastomas) OR abstract:(hepatoblastomas)) OR (title:(liver carcinogenesis) OR abstract:(liver carcinogenesis)) OR (title:(morris hepatomas) OR abstract:(morris hepatomas)) OR		

	(title:(novikoff hepatomas) OR abstract:(novikoff hepatomas))))		
#2	(title:(Liver transplantation) OR abstract:(Liver transplantation))		**Search terms:** Liver transplantation
#3	#1 AND #2	1,515	Combine search terms

Table S2: Reasons for excluded studies

Study ID	Title	Included/Excluded	Reason for Exclusion
A. A. M. Al-Ameri et al. 2019	Preoperative risk stratification for early recurrence of HBV-related hepatocellular carcinoma after deceased donor liver transplantation: a five-eight model development and validation	Excluded	Irrelevant Subgroup
S. F. Ang et al. 2015	The Singapore Liver Cancer Recurrence (SLICER) Score for relapse prediction in patients with surgically resected hepatocellular carcinoma	Excluded	Intervention irrelevant
U. Baccarani et al. 2008	Superiority of transplantation versus resection for the treatment of small hepatocellular carcinoma	Excluded	Irrelevant Sub group
J. F. Castroagudín et al. 2009	Safety of an immunosuppressant protocol based on sirolimus in liver transplant recipients with malignancies or high risk of tumor recurrence	Excluded	Irrelevant Sub group
S. Chinnakotla et al. 2009	Impact of sirolimus on the recurrence of hepatocellular carcinoma after liver transplantation	Excluded	Irrelevant Sub group
O. Detry et al. 2015	Prognostic value of (18)F-FDG PET/CT in liver transplantation for hepatocarcinoma	Excluded	Outcome irrelevant
S. Dumitra et al. 2013	Hepatitis C infection and hepatocellular carcinoma in liver transplantation: a 20-year experience	Excluded	Irrelevant Sub group
S. T. Fan et al. 2009	Follow-up of Chinese liver transplant recipients in Hong Kong	Excluded	Irrelevant Sub group
J. Figueras et al. 1997	Survival after liver transplantation in cirrhotic patients with and without hepatocellular carcinoma: a comparative study	Excluded	study design irrelevant
J. Fujimoto et al. 1993	Efficacy of autotransfusion in hepatectomy for hepatocellular carcinoma	Excluded	Outcome irrelevant
A. E. Giakoustidis et al. 2017	Immunosuppression strategies in liver transplantation patient; patients with hepatocellular carcinoma	Excluded	Outcome irrelevant
B. Hu et al. 2014	Systemic immune-inflammation index predicts prognosis of patients after curative resection for hepatocellular carcinoma	Excluded	population irrelevant
Z. Hu et al. 2012	Recipient outcomes of salvage liver transplantation versus primary liver transplantation: a systematic review and meta-analysis	Excluded	study design irrelevant

Study ID	Title	Included/Excluded	Reason for Exclusion
Z. Hu et al. 2015	Impact of multiple liver resections prior to salvage liver transplantation on survival in patients with recurrent HCC	Excluded	Population Irrelevant
W. H. Kang et al. 2017	Prognostic effect of transarterial chemoembolization-induced complete pathological response in patients undergoing liver resection and transplantation for hepatocellular carcinoma	Excluded	Irrelevant Sub group
D. J. Kim et al. 2014	Recurrence of hepatocellular carcinoma: importance of mRECIST response to chemoembolization and tumor size	Excluded	study design irrelevant
J. M. Kim et al. 2014	Expanded criteria for liver transplantation in patients with hepatocellular carcinoma	Excluded	Outcome irrelevant
V. Kohli et al. 2012	Antiviral therapy for recurrent hepatitis C reduces recurrence of hepatocellular carcinoma following liver transplantation	Excluded	Irrelevant Sub group
A. Kornberg et al. 2009	Increased 18F-FDG uptake of hepatocellular carcinoma on positron emission tomography independently predicts tumor recurrence in liver transplant patients	excluded	Outcome irrelevant
A. Kornberg et al. 2013	Postinterventional tumor necrosis predicts recurrence-free long-term survival in liver transplant patients with advanced hepatocellular carcinoma	excluded	Outcome irrelevant
A. Lauterio et al. 2010	Hepatocellular carcinoma in unrelated viral cirrhosis: long-term results after liver transplantation	Excluded	Outcome irrelevant
E. C. Lee et al. 2016	High-dose hepatitis B immunoglobulin therapy in hepatocellular carcinoma with hepatitis B virus-DNA/hepatitis B e antigen-positive patients after living donor liver transplantation	Excluded	Outcome irrelevant
J. Lei et al. 2013	Comparison of the outcomes of patients who underwent deceased-donor or living-donor liver transplantation after successful downstaging therapy	Excluded	Irrelevant Sub group
D. Lu et al. 2018	The association between donor genetic variations in one-carbon metabolism pathway genes and hepatitis B recurrence after liver transplantation	Excluded	Outcome Irrelevant
V. R. Mas et al. 2007	Genes associated with progression and recurrence of hepatocellular carcinoma in hepatitis C patients waiting and undergoing liver transplantation: preliminary results	Excluded	Outcome irrelevant
V. Mazzaferro et al. 2018	Metroticket 2.0 Model for Analysis of Competing Risks of Death After Liver Transplantation for Hepatocellular Carcinoma	Excluded	Irrelevant Sub group

Study ID	Title	Included/Excluded	Reason for Exclusion
J. Michel et al. 1995	Recurrence of hepatocellular carcinoma in cirrhotic patients after liver resection or transplantation	Excluded	Not available
J. Michel et al. 1997	Liver resection or transplantation for hepatocellular carcinoma? Retrospective analysis of 215 patients with cirrhosis	Excluded	Irrelevant Sub group
T. Miyata et al. 2022	The Impact of Histologic Liver Inflammation on Oncology and the Prognosis of Patients Undergoing Hepatectomy for Hepatocellular Carcinoma	Excluded	Not available
P. Moreno et al. 1995	Orthotopic liver transplantation: treatment of choice in cirrhotic patients with hepatocellular carcinoma?	Excluded	Not available
M. Murawski et al. 2016	Hepatocellular Carcinoma in Children: Does Modified Platinum- and Doxorubicin-Based Chemotherapy Increase Tumor Resectability and Change Outcome? Lessons Learned From the SIOPEL 2 and 3 Studies	Excluded	Study design irrelevant
H. Nafady-Hego et al. 2016	Outcome of Hepatitis B Virus Infection After Living-Donor Liver Transplant: A Single-center Experience Over 20 Years	Excluded	Population irrelevant
Y. Nagaoki et al. 2019	The impact of interferon-free direct-acting antivirals on clinical outcome after curative treatment for hepatitis C virus-associated hepatocellular carcinoma: Comparison with interferon-based therapy	Excluded	Population irrelevant
A. Nanashima et al. 2010	Relationship between period of survival and clinicopathological characteristics in patients with hepatocellular carcinoma who underwent hepatectomy	Excluded	Population irrelevant
P. Neuhaus et al. 1999	Liver transplantation for hepatocellular carcinoma	Excluded	Publication type irrelevant
D. Nobuoka et al. 2010	Postoperative serum alpha-fetoprotein level is a useful predictor of recurrence after hepatectomy for hepatocellular carcinoma	Excluded	Population irrelevant
Y. Okuda et al. 2014	Clinicopathological factors affecting survival and recurrence after initial hepatectomy in non-B non-C hepatocellular carcinoma patients with comparison to hepatitis B or C virus	Excluded	Population irrelevant
G. Orlando et al. 2013	Hepatic hemangiosarcoma: an absolute contraindication to liver transplantation--the European Liver Transplant Registry experience	Excluded	Population irrelevant

Study ID	Title	Included/Excluded	Reason for Exclusion
G. Otto et al. 2006	Response to transarterial chemoembolization as a biological selection criterion for liver transplantation in hepatocellular carcinoma	Excluded	Population irrelevant
P. M. Rizzi et al. 1994	Neoadjuvant chemotherapy after liver transplantation for hepatocellular carcinoma	Excluded	Not available
S. Saab et al. 2010	Sorafenib as adjuvant therapy for high-risk hepatocellular carcinoma in liver transplant recipients: feasibility and efficacy	Excluded	Not available
S. Saab et al. 2009	Recurrence of hepatocellular carcinoma and hepatitis B reinfection in hepatitis B surface antigen-positive patients after liver transplantation	Excluded	Population irrelevant
M. I. Sánchez-Lorencio et al. 2018	Matrix Metalloproteinase 1 as a Novel Biomarker for Monitoring Hepatocellular Carcinoma in Liver Transplant Patients	Excluded	Outcome irrelevant
G. Sapisochin et al. 2011	Mixed hepatocellular cholangiocarcinoma and intrahepatic cholangiocarcinoma in patients undergoing transplantation for hepatocellular carcinoma	Excluded	Irrelevant Sub group
Y. Sato et al. 2005	Preoperative human-telomerase reverse transcriptase mRNA in peripheral blood and tumor recurrence in living-related liver transplantation for hepatocellular carcinoma	Excluded	Not available
O. Scatton et al. 2008	Hepatocellular carcinoma developed on compensated cirrhosis: resection as a selection tool for liver transplantation	Excluded	Irrelevant Sub group
M. Schwartz et al. 2005	How should patients with hepatocellular carcinoma recurrence after liver transplantation be treated?	Excluded	Study design irrelevant
Z. Shao et al. 2008	Orthotopic liver transplantation as a rescue operation for recurrent hepatocellular carcinoma after partial hepatectomy	Excluded	Irrelevant Sub group
I. S. Sheen et al. 2005	Do the expressions of gap junction gene connexin messenger RNA in noncancerous liver remnants of patients with hepatocellular carcinoma correlate with postoperative recurrences?	Excluded	Population irrelevant
G. C. Sotiropoulos et al. 2008	Single-center experience on liver transplantation for hepatocellular carcinoma arising in alcoholic cirrhosis: results and ethical issues	Excluded	Study design irrelevant
G. C. Sotiropoulos et al. 2006	De novo hepatocellular carcinoma in recurrent liver cirrhosis after liver transplantation for benign hepatic disease: is a deceased donor re-transplantation justified?	Excluded	Study design irrelevant

Study ID	Title	Included/Excluded	Reason for Exclusion
T. E. Starzl et al. 1978	Surgical approaches for primary and metastatic liver neoplasms, including total hepatectomy with orthotopic liver transplantation	Excluded	Population irrelevant
L. Y. Sun et al. 2014	Prognosis in liver transplantation recipients after hepatitis B virus recurrence	Excluded	Not available
M. Tonolini et al. 2002	Extrahepatic recurrence and second malignancies after treatment of hepatocellular carcinoma: spectrum of imaging findings	Excluded	Not available
A. D. Trobaugh-Lotrario et al. 2016	Outcomes of Patients With Relapsed Hepatoblastoma Enrolled on Children's Oncology Group (COG) Phase I and II Studies	Excluded	Population irrelevant
Y. F. Tsai et al. 2016	Nationwide population-based study reveals increased malignancy risk in taiwanese liver transplant recipients	Excluded	Outcome irrelevant
D. I. Tsilimigras et al. 2020	Recurrence Patterns and Outcomes after Resection of Hepatocellular Carcinoma within and beyond the Barcelona Clinic Liver Cancer Criteria	Excluded	Population irrelevant
S. Tsukamoto et al. 2014	Survival after resection of liver and lung colorectal metastases in the era of modern multidisciplinary therapy	Excluded	Population irrelevant
J. Tu et al. 2015	Effectiveness of combined (131)I-chTNT and radiofrequency ablation therapy in treating advanced hepatocellular carcinoma	Excluded	Population irrelevant
K. Uchino et al. 2018	Serum levels of ferritin do not affect the prognosis of patients with hepatocellular carcinoma undergoing radiofrequency ablation	Excluded	Population irrelevant
S. Uemura et al. 2014	ERCC1 mRNA expression as a postoperative prognostic marker in extrahepatic bile duct cancer	Excluded	Population irrelevant
H. Ueno et al. 2004	Predictors of extrahepatic recurrence after resection of colorectal liver metastases	Excluded	Population irrelevant
K. Umeda et al. 2018	Prognostic and therapeutic factors influencing the clinical outcome of hepatoblastoma after liver transplantation: A single-institute experience	Excluded	Population irrelevant
K. Vakili et al. 2009	Living donor liver transplantation for hepatocellular carcinoma: Increased recurrence but improved survival	Excluded	Irrelevant Sub group
A. Van Der Gucht et al. 2017	Resin Versus Glass Microspheres for (90)Y Transarterial Radioembolization: Comparing Survival in Unresectable Hepatocellular Carcinoma Using Pretreatment Partition Model Dosimetry	Excluded	Population irrelevant
E. P. van der Stok et al. 2016	mRNA expression profiles of colorectal liver metastases as a novel biomarker for early recurrence after partial hepatectomy	Excluded	Population irrelevant

Study ID	Title	Included/Excluded	Reason for Exclusion
M. A. Varona et al. 2015	Risk factors of hepatocellular carcinoma recurrence after liver transplantation: accuracy of the alpha-fetoprotein model in a single-center experience	Excluded	Not available
B. Vasavada et al. 2016	Prior Trans-arterial chemoembolization - A protective factor against rapid HCV recurrence post liver transplant in patients with HCV with HCC? - A Retrospective Cohort Study	Excluded	Outcome irrelevant
B. B. Vasavada et al. 2015	Rapid fibrosis and significant histologic recurrence of hepatitis C after liver transplant is associated with higher tumor recurrence rates in hepatocellular carcinomas associated with hepatitis C virus-related liver disease: a single center retrospective analysis	Excluded	Not available
M. A. Veenstra et al. 2016	Minimally-invasive liver resection in pediatric patients: initial experience and outcomes	Excluded	Population irrelevant
E. Virgilio et al. 2016	Port Site Recurrences Following Laparoscopic Liver Resection for Hepatocellular Carcinoma	Excluded	Study design irrelevant
M. Vivarelli et al. 2010	Sirolimus in liver transplant recipients: a large single-center experience	Excluded	Outcome irrelevant
T. Wakai et al. 2002	Computed tomographic features of hepatocellular carcinoma predict long-term survival after hepatic resection	Excluded	Not available
T. Wakai et al. 2001	Early gallbladder carcinoma does not warrant radical resection	Excluded	Population irrelevant
K. Wakayama et al. 2017	Huge hepatocellular carcinoma greater than 10 cm in diameter worsens prognosis by causing distant recurrence after curative resection	Excluded	Outcome irrelevant
S. Wan et al. 2015	Polymorphisms in Genes of Tricarboxylic Acid Cycle Key Enzymes Are Associated with Early Recurrence of Hepatocellular Carcinoma	Excluded	Population irrelevant
B. Wang et al. 2020	Tfr-Tfh index: A new predicator for recurrence of hepatocellular carcinoma patients with HBV infection after curative resection	Excluded	Population irrelevant
H. Wang et al. 2021	Postoperative adjuvant transcatheter arterial chemoembolization improves the prognosis of patients with huge hepatocellular carcinoma	Excluded	Population irrelevant
L. Wang et al. 2015	Identification of recurrence-related serum microRNAs in hepatocellular carcinoma following hepatectomy	Excluded	Population irrelevant
Q. Wang et al. 2016	Impact of non-neoplastic vs intratumoural hepatitis B viral DNA and replication on hepatocellular carcinoma recurrence	Excluded	Population irrelevant

Study ID	Title	Included/Excluded	Reason for Exclusion
X. Wang et al. 2016	Combined measurements of tumor number and size helps estimate the outcome of resection of Barcelona clinic liver cancer stage B hepatocellular carcinoma	Excluded	Population irrelevant
K. N. Wanis et al. 2018	Intermediate-term survival and quality of life outcomes in patients with advanced colorectal liver metastases undergoing associating liver partition and portal vein ligation for staged hepatectomy	Excluded	Population irrelevant
T. Wei et al. 2021	Early Versus Late Recurrence of Hepatocellular Carcinoma After Surgical Resection Based on Post-recurrence Survival: an International Multi-institutional Analysis	Excluded	Population irrelevant
P. Y. Wong et al. 1995	Clinical course and survival after liver transplantation for hepatitis B virus infection complicated by hepatocellular carcinoma	Excluded	Outcome irrelevant
D. Xuan et al. 2021	Survival comparison between radiofrequency ablation and surgical resection for patients with small hepatocellular carcinoma: A systematic review and meta-analysis	Excluded	Population irrelevant
Y. Yamashita et al. 2013	Third or more repeat hepatectomy for recurrent hepatocellular carcinoma	Excluded	Population irrelevant
J. D. Yang et al. 2016	Direct acting antiviral therapy and tumor recurrence after liver transplantation for hepatitis C-associated hepatocellular carcinoma	Excluded	Irrelevant Sub group
S. Yilmaz et al. 2021	The Importance of the Immunosuppressive Regime on Hepatocellular Carcinoma Recurrence After Liver Transplantation	Excluded	Not available
L. P. Zanaga et al. 2017	Survival benefits of interferon-based therapy in patients with recurrent hepatitis C after orthotopic liver transplantation	Excluded	Outcome irrelevant
J. Zhang et al. 2016	Prospective, single-center cohort study analyzing the efficacy of complete laparoscopic resection on recurrent hepatocellular carcinoma	Excluded	Population irrelevant
X. F. Zhang et al. 2014	Impact of cigarette smoking on outcome of hepatocellular carcinoma after surgery in patients with hepatitis B	Excluded	Population irrelevant
Y. T. Zhang et al. 2015	Vincristine and irinotecan in children with relapsed hepatoblastoma: a single-institution experience	Excluded	Population irrelevant
Y. M. Zhou et al. 2011	Safety and efficacy of partial hepatectomy for huge (≥10 cm) hepatocellular carcinoma: a systematic review	Excluded	Population irrelevant
G. Q. Zhu et al. 2015	Optimal adjuvant therapy for resected hepatocellular carcinoma: a systematic review with network meta-analysis	Excluded	Population irrelevant

Study ID	Title	Included/Excluded	Reason for Exclusion
J. Zhu et al. 2011	CEACAM1 cytoplastic expression is closely related to tumor angiogenesis and poorer relapse-free survival after curative resection of hepatocellular carcinoma	Excluded	Population irrelevant
Z. Hu et al. 2014	Salvage liver transplantation for recurrent hepatocellular carcinoma after liver resection: retrospective study of the Milan and Hangzhou criteria	Excluded	Outcome irrelevant
B. P. Saborido et al. 2005	Does preoperative fine needle aspiration-biopsy produce tumor recurrence in patients following liver transplantation for hepatocellular carcinoma?	Excluded	Outcome irrelevant
K. Staufer et al. 2012	High toxicity of sorafenib for recurrent hepatocellular carcinoma after liver transplantation	Excluded	Outcome irrelevant
C. Zavaglia et al. 2013	Adverse events affect sorafenib efficacy in patients with recurrent hepatocellular carcinoma after liver transplantation: experience at a single center and review of the literature	Excluded	Outcome irrelevant

Table S3: Characteristics of included studies

S. No	Author	Year	Country	Sample size	Mean Age (years)	Male (%)
1	Ataide et al.	2011	Brazil	83	55.3	82
2	Alvite-Canosa et al.	2010	Spain	150	56.4	88
3	Baskiran et al.	2020	Turkey	194	NR	NR
4	Bassanello et al.	2003	Italy	48	52*	83.3
5	Bernal et al.	2006	Spain	12	48	92
6	Bilbao et al.	2009	Spain	25	55.6	76
7	Campsen et al.	2013	USA	354	51.8	81.6
8	Chagas et al.	2019	Brazil	1119	58	66.9
9	Chan et al.	2020	China	265	49*	88.9
10	Chao et al.	2007	San Francisco	100	55.5	71
11	Chung et al.	1994	France	125	52.6*	85.6
12	D'Amico et al.	2009	Italy	479	56.8	78
13	Revilla et al.	2003	USA	10	55	100
14	Martin et al.	2008	Italy	71	54*	70.8
15	Escartin et al.	2007	Spain	202	59	73.7
16	Fan et al.	2009	China	1078	49*	88.9
17	Farinati et al.	2001	Italy	4	63.4*	65
18	Fernandez-Sevilla et al.	2017	France	493	55*	87.1
19	Ferris et al.	1996	USA	124	54	80
20	Foltys et al.	2014	Germany	41	61.3*	82.4

S. No	Author	Year	Country	Sample size	Mean Age (years)	Male (%)
21	Gonzalez-Uriarte et al.	2003	USA	88	58*	77.2
22	Ito et al.	2007	Japan	125	55*	71.2
23	Kim et al.	2011	South Korea and China	132	55*	89.7
24	Kiyici et al.	2008	Turkey	72	52.3	84.7
25	Kornberg et al.	2009	Germany	42	61*	83.3
26	Kornberg et al.	2011	Germany	60	58.2	76.6
27	Kornberg et al.	2018	Germany	119	58.5	59.6
28	Kornberg et al.	2015	Germany	103	58.6	62.1
29	Lai et al.	2013	Belgium, Italy	422	60*	82.4
30	Lee et al.	2005	South Korea	87	NR	NR
31	Lei et al.	2013	China	31	43	64.5
32	Li et al.	2011	China	148	47.1	86.7
33	Llovet et al.	1998	Spain	58	52.4	81
34	Macaron et al.	2010	USA	107	55.9*	86.9
35	Marsh et al.	1998	USA	214	50.4	74.7
36	Ares et al.	2004	Spain	80	52.6	81.2
37	Marubashi et al.	2006	USA	25	NR	72
38	Miyagi et al.	2012	Japan	14	53.1	64.2
39	Northen et al.	2018	Germany	63	59.6	84.1
40	Otto et al.	2007	Germany	60	60.1*	76.6
41	Otto et al.	1997	Germany	50	45.8	80
42	Quan et al.	2006	UK	75	53.5	84
43	Park et al.	2006	Korea	156	50.1*	95.6
44	Luque et al.	2006	Spain	81	53.8	88.9
45	Pfiffer et al.	2011	Germany	139	57.8	95.8
46	Philosophe et al.	1998	Canada	60	56*	NR
47	Ramos et al.	2006	Spain	67	60	61.1
48	Rayya et al.	2008	Germany	11	56*	81.8
49	Regalia et al.	1997	Italy	132	50	15.9
50	Sa et al.	2016	Brazil	414	56	79.5
51	Samoylova et al.	2014	USA	5002	57*	77.4
52	Sandow et al.	2018	New Orlean	82	59.6	78.4
53	Santoyo et al.	2009	Spain	186	NR	NR
54	Santoyo et al.	2005	Spain	53	57	81.3
55	Sauer et al.	2005	Germany	110	NR	#VALUE!
56	Schraiber et al.	2016	Brazil	206	56.3	74.7
57	Sha et al.	2012	China	48	NR	91.6
58	Sieghart et al.	2011	Austria	125	54.5	84.8
59	Silva et al.	2008	Spain	257	60*	80.5
60	Taketomi et al.	2010	Japan	101	NR	71.2
61	Tan et al.	1995	UK	15	NR	NR

S. No	Author	Year	Country	Sample size	Mean Age (years)	Male (%)
62	Triana et al.	2016	Spain	10	11.5	80
63	Varona et al.	2009	Spain	61	55*	80
64	Vatansever et al.	2019	Turkey	54	55.6	92.5
65	Vigano et al.	2015	Italy	101	54*	94
66	Vitale et al.	2012	Italy	165	NR	NR
67	Wan et al.	2014	China	130	49.5	86.1
68	Wan et al.	2014	China	226	50.2	84.9
69	Xu et al.	2009	China	97	47.9	89.69072
70	Yi et al.	2007	Korea	85	NR	NR
71	Yokoyama et al.	1991	USA	106	43.4	66.9
72	Zimmerman et al.	2007	USA	100	56	81
73	Diniz et al.	2020	Brazil	127	57.7	80
74	Morgul et al.	2020	Germany	120	61	81.7
75	Pravisani et al.	2020	Italy	280	57	89.6
76	Yang et al.	2020	China	62	52	90
77	Ismael et al.	2019	USA	160	58	78.1
78	Kang et al.	2019	Korea	239	56	90
79	Lee et al.	2019	Korea	122	54	84.4
80	Mahmud et al.	2019	USA	18406	57.5	78.5
81	Polat et al.	2019	Turkey	165	57.7	80
82	Zeair et al.	2019	Poland	166	58	74.6
83	Zeng et al.	2019	China	99	48.4	90
84	Foerster et al.	2018	Germany	304	59.9	77.3
85	Mehta et al.	2018	USA	740	58	74.2
86	Pinna et al.	2018	Italy, China	1218	53.7	86.6
87	Sadler et al.	2018	USA	929	63.1	61.7
88	Valverde-Lopez et al.	2018	Spain	89	57.9	69
89	Yilmaz et al.	2018	Turkey	187	55.3	86.6
90	Halazun et al.	2017	USA	339	57.8	80
91	Kamel et al.	2017	Egypt	60	52	93
92	Kositamongkol et al.	2017	Thailand	63	54	95
93	Pinero et al.	2017	Latin America	435	55	73
94	Azoulay et al.	2016	France	651	57	81.7
95	Boteon et al.	2016	Brazil	101	55.8	86.1
96	Colhoun et al.	2016	USA	141	58	78
97	Donat et al.	2016	Spain	151	54.5	78.1
98	Grat et al.	2016	Poland	240	57	72
99	Irtan et al.	2016	France	179	54	88.8
100	Yang et al.	2016	Korea	88	59	81
101	Macdonald et al.	2015	USA	1074	57.5	75
102	Orci et al.	2015	Switzerland	9724	57	75
103	Costa et al.	2014	Brazil	140	56.5	82.1

S. No	Author	Year	Country	Sample size	Mean Age (years)	Male (%)
104	Squires III et al.	2014	USA	131	59	73.5
105	Wong et al.	2014	USA	114	61.3	77.5
106	Choi et al.	2013	Korea	224	50.8	89.2
107	Doyle et al.	2012	USA	264	55.9	79
108	Sharma et al.	2012	USA	94	54.5	71
109	Chan et al.	2011	Taiwan	126	50.5	78
110	Chok et al.	2011	Hong Kong, China	139	49.5	98
111	Kaido et al.	2011	Japan	164	56	65
112	Lai et al.	2011	Italy	153	57	83
113	Coelho et al.	2009	Brazil	45	56	79
114	McHugh et al.	2009	USA	101	55.7	79
115	Castroagudin et al.	2008	Spain	130	57.8	81
116	Lee et al.	2008	Korea	221	51	79.2
117	Malago et al.	2006	Germany	34	54	75
118	Parfitt et al.	2006	Canada	75	57.5	89
119	Island et al.	2005	USA	92	53	80
120	Merli et al.	2005	Italy	63	54	80
121	Roayaie et al.	2004	US	311	56	
122	Todo et al.	2004	Japan	316	59	86
123	De Carlis et al.	2003	Italy	99	51	80
124	Vivarelli et al.	2002	Italy	82	56	85
125	Chui et al.	1999	Hong Kong, China	24	49	98

*: Median; NR: Not Reported; USA: United States of America; UK: United Kingdom
